# Assessment of Ecological Bridges at Wildlife Crossings in Türkiye: A Case Study of Wild Boar Crossings on the Izmir-Çeşme Motorway

**DOI:** 10.3390/ani14010030

**Published:** 2023-12-21

**Authors:** Uğur Tuttu, Efehan Ulaş, Derya Gülçin, Javier Velázquez, Kerim Çiçek, Ali Uğur Özcan

**Affiliations:** 1 Department of Wildlife, Institute of Natural and Applied Sciences, Çankırı Karatekin University, Çankırı 18200, Türkiye; ugur.tuttu@gmail.com (U.T.); auozcan@karatekin.edu.tr (A.U.Ö.); 2 Department of Statistics, Science Faculty, Çankırı Karatekin University, Çankırı 18200, Türkiye; 3 Faculty of Agriculture, Department of Landscape Architecture, Aydın Adnan Menderes University, Aydın 09100, Türkiye; 4 TEMSUS Research Group, Catholic University of Ávila, 05005 Ávila, Spain; javier.velazquez@ucavila.es (J.V.); kerim.cicek@ege.edu.tr (K.Ç.); 5 Faculty of Sciences and Arts, Department of Environment and Agroforestry, Catholic University of Ávila, 05005 Ávila, Spain; 6 Tecnatura Research Group, Technical University of Madrid, 28040 Madrid, Spain; 7 Faculty of Science, Department of Biology, Section of Zoology, Ege University, Izmir 35100, Türkiye; 8 Natural History Application and Research Centre, Ege University, Izmir 35100, Türkiye; 9 Faculty of Forestry, Department of Landscape Architecture, Çankırı Karatekin University, Çankırı 18200, Türkiye

**Keywords:** wildlife–vehicle collision, wildlife overpass, environmental monitoring, fragmentation

## Abstract

**Simple Summary:**

This study evaluates the effectiveness of an ecological bridge, constructed in 2020 in the Zeytinler neighborhood, as a solution to mitigate wild-boar–vehicle collisions (WVCs) on the Izmir-Çeşme motorway. The Zeytinler Ecological Bridge was monitored and analyzed for wildlife crossings, particularly by wild boars. Between August 2020 and December 2022, 686 instances of movement were observed among six wild mammal species, with wild boars representing 87.5% and foxes 10% of the recorded crossings. The study indicates that the highest frequency of wildlife crossings occurred in autumn between 22:00 and 02:00, coinciding with specific moon phases. Moreover, wild boar crossings increased during the autumn season with a full pond, and no wild boar fatalities were recorded after the bridge’s completion. The findings suggest that the ecological bridge effectively facilitates safe wildlife passage, especially for wild boars, reducing the risk of collisions with vehicles on the motorway.

**Abstract:**

In this study, the use of an ecological bridge installed as a wildlife overpass and constructed in the Zeytinler neighborhood in 2020 was analyzed as a mitigating factor in wild-boar–vehicle collisions (WVCs) on the Izmir-Çeşme motorway. In this context, this study aimed to assess the use of the Zeytinler Ecological Bridge by wild boars (*Sus scrofa* Linnaeus, 1758). To this end, wildlife crossings were monitored, analyzed, and modeled with Bayesian networks. Between August 2020 and December 2022, a total of 686 instances of movement were observed among six medium to large wild mammal species. Wild boars accounted for approximately 87.5% of the recorded wildlife crossings, with foxes comprising 10%. The findings showed that the highest frequency of wildlife crossings occurred during the autumn season, particularly between 22:00 (10 p.m.) and 02:00 (2 a.m.), coinciding with the Waxing Gibbous and Waxing Crescent phases of the moon. The model outcomes highlighted that during the autumn season with a full pond, wild boar crossings increased by one and a half times in comparison to regular herd crossings. Throughout the observation period, there were no instances of wild boar fatalities subsequent to the completion of the bridge.

## 1. Introduction

Transportation infrastructures disrupt natural habitats, acting as a barrier that hinders the movement of wildlife across the landscape [1,2]. This often leads to animals either being unable to traverse these structures or actively avoiding them [3]. Consequently, it results in wildlife–vehicle collisions (WVCs) and ultimately diminishes the connectivity between different landscape patches [4,5]. Since roads and their associated networks have the potential to influence species gene flow [6,7], WVCs pose a threat to the enduring existence of wildlife populations, communities, and ecosystems [8,9]. In addition, the degradation of habitats and reduction in patch sizes are leading to increased local extinctions of small populations, and the decrease in connectivity is leading to less colonization of patches [10,11,12,13]. 

Animals may react to roads in different ways, as well as avoiding or retreating from them [14,15]. Their response can be associated with the road effect [16,17]. The road effect represents the distance from the road edge where significant ecological impacts become apparent [1]. Some animals perceive roads as barriers and refrain from attempting to cross them. For instance, one study observed that the road effect had a notably adverse impact on the species richness and relative abundance of amphibians, affecting four out of seven species within a range of 250–1000 m from the road edge [18]. Another study found that the abundance of forest and pasture bird species indicated a significant decrease in diversity and breeding activities at distances ranging from 300 to 1000 m from the roads [19].

WVCs represent one of the most conspicuous and adverse outcomes of the transportation network [20,21]. Extensive research on WVC patterns has demonstrated that these collisions are not random events but tend to cluster spatially [5,22,23,24,25]. Studies focusing on the causes of WVC have typically unveiled the influence of traffic characteristics, highway attributes, and habitat usage, employing diverse statistical models [26,27,28]. A literature review conducted by Gunson et al. [27] uncovered that the presence of forest and open habitats near roads tends to elevate the frequency of ungulate collisions. Conversely, the proximity of agricultural and urban areas to roads has been associated with a reduction in the number of such collisions. Moreover, WVCs typically reached their peak occurrence on road stretches marked by elevated traffic volumes, reduced visibility for motorists, and cases where roads intersected with drainage movement corridors or traversed level terrain [27,29]. However, the relationships are not always linear, and WVCs may peak on roads with medium traffic volume [30]. For instance, recent studies conducted in Türkiye showed that WVCs occur more in medium traffic volumes than in low and high traffic volumes [5,25]. 

Numerous precautions are necessary to ensure the safe passage of wild animals through transportation networks. These measures encompass a range of strategies, including the installation of wildlife warning signs, efforts to reduce traffic volume and speed, implementation of animal detection systems, use of wildlife reflectors and repellents, modification of road designs, viaducts, and bridges, adjustments in roadside management, installation of wildlife fencing, establishment of wildlife pedestrian passages, and the creation of wildlife crossing structures [31,32,33]. Implementing and maintaining wildlife fencing and crossing structures in accordance with the specific needs of target species can lead to a significant reduction in large mammal–vehicle collisions, ranging from 80% to 97% [34,35]. Wildlife crossing structures (WCS), often referred to simply as “wildlife crossings” or “ecological passages”, are specialized infrastructure designed to facilitate the safe movement of wildlife across roads and highways. These structures are created to address the challenges posed by transportation networks, which can fragment natural habitats, disrupt animal crossing patterns, and increase the risk of wildlife–vehicle collisions. The effectiveness of these WCS is directly tied to their strategic placement and design [36]. To mitigate wildlife accidents, decision makers must carefully consider various factors in the decision-making process, as the design and implementation of ecological bridges entail significant economic costs. The responsible institution is in the planning stages of constructing a wildlife overpass in response to the rising number of traffic accidents on the Izmir-Çesme motorway, attributed to wild boars crossing the road and breaking through wires at some locations. The primary objective behind the construction of an ecological bridge is to mitigate WVCs. In this context, a wildlife crossing in an ecological bridge designed as a wildlife overpass, was monitored to assess whether WVCs were mitigated and to understand crossing in a fragmented landscape. The monitoring system was installed immediately when the ecological bridge opened in July 2020. Here, the target species is wild boars since the bridge was constructed to sustain the ecological connectivity of large mammals. To this end, wildlife crossings were analyzed and modeled with the Bayesian network model. Here, three primary questions were explored:

(1) What is the temporal distribution of wild boar crossings on the Ecological Bridge?
(2) When do wild boars cross the motorway and how does the presence of the incorporated pond on the bridge affect these crossings?
(3) What measures should be considered for the effective use of the wildlife crossing and the implementation of collision mitigation strategies?

This study not only examined the function of the ecological bridge for wildlife but also offered valuable insights into understanding wildlife crossings. The result of the study led to the development of an adaptable guide for future overpasses, specifically addressing target wildlife species and design improvements.

## 2. Material and Methods

### 2.1. Study Area

**Figure 1:**
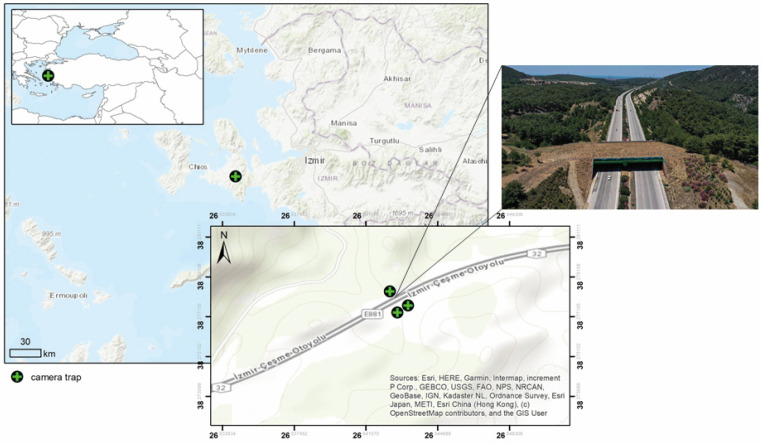
The location of study area, camera traps, and general view of the Zeytinler Ecological Bridge.

The study area encompasses the Çeşme-Urla Peninsula, located in the westernmost region of Türkiye and divided by the Izmir-Çeşme motorway (Figure 1). Encompassing an approximate area of 1379 km^2^, this region incorporates the districts of Karaburun, Urla, and Çeşme. The land usage of both sides of the ecological bridge is similar. Agricultural areas and forests are dominant land uses. The peninsula hosts three distinct vegetation formations: forests, maquis, and phrygana [37]. The dominant overstory species is the red pine (*Pinus brutia* Ten.). Some types of maquis vegetation encroach into the brutian stands due to their ability to reach sufficient levels of light. Kermes oak (*Quercus coccifera* L.) is a key species, followed by carob (*Calycotome villosa (Poir.) Link*), cade juniper (*Juniperus oxycedrus* L.), sage-leaved rock rose (*Cistus salviifolius* L.), and sandalwood (*Arbutus andrachne* L.). Other dominant species are mock privet (*Phillyrea latifolia* L.), tree heath (*Erica arborea* L.), storax (*Styrax officinalis* L.), and mastic tree (*Pistacia lentiscus* L.) [37].

The Çeşme-Karaburun peninsulas have been identified as particularly vulnerable to deforestation. Research in Karaburun has indicated that garrigue vegetation has spread extensively due to deforestation. Agricultural activities are prevalent in the non-forested areas, with Uzunkuyu and Nohutalan Districts to the north of the bridge and the Zeytineli District to the south, with the cultivation of citrus fruits, olives, viticulture, and grain products [38,39,40]. As of 2019, the total population of the three districts on the peninsula was 135.860 individuals [41]. The population quadruples in the summer months. For instance, 70% of the 55.733 houses in Çeşme are used for 3–4 months a year [42]. 

Based on the Köppen climate classification, the air temperature within the study area fluctuates between 1 °C and 33 °C, with the peak temperature reaching 33 °C, indicative of a climate predominantly characterized by “ warm winters, very hot summers, and arid conditions” [43]. The average highest daily temperature exceeds 18 °C. During August, the warmest month, the average maximum temperature reaches 32 °C, while the lowest temperature remains around 25 °C. January represents the coldest month in the study area, with average temperatures ranging from 1 °C to 19 °C. While the summers are hot and dry in the study area, the winter months are warm and rainy. The average daily precipitation measures 1.4 mm, although the highest recorded precipitation level reaches 62.3 mm. 

In the study area, there are medium and large mammal species such as golden jackal (*Canis aureus* Linnaeus, 1758), red fox (*Vulpes vulpes* Linnaeus, 1758), wild boar (*Sus scrofa* Linnaeus, 1758), and European badger (*Meles meles* Linnaeus, 1758). In their ecological network study conducted for Izmir, Hepcan et al. [44,45] identified the caracal (*Caracal caracal* Schreber, 1776) and striped hyena (*Hyaena hyaena* Linnaeus, 1758) as the target species. However, there has been no documented presence of the caracal in the study area for the past decade or the striped hyena for the last 40 years. Consequently, the largest mammal species in the study area is the wild boar. 

The Izmir-Çeşme motorway is surrounded by a fence, has six lanes in both directions, and is 77.7 km long. The traffic volume of the highway is moderate (approximately 11.000 vehicle/day). Here, the speed limit is 130 km/h. To reduce WVCs involving wild boars, wildlife overpasses were constructed at three distinct locations along the Izmir-Ceşme motorway. The initial overpass was opened in Zeytinler in 2020, with plans for the second overpass to be operational by 2024. In the site selection process, the recommendations of the General Directorate of Highways (KGM) and traffic control units, along with the identification of locations with concentrated wild boar collision incidents, were considered. Previously, when the Zeytinler Ecological Bridge was built, the suitability of the location was technically evaluated but not ecologically planned. The results of this study will contribute to the positioning of the new bridge planned for 2024. With a total length of 64 m and an average width of 52 m, the narrowest section of the bridge is 40 m; the bridge is supported by side retaining walls. During the construction process, 2.5 m of soil fill was incorporated for the upper design, along with the planting of 2.200 trees and shrubs. Additionally, a concrete pond, 4 m in diameter and 50 cm deep, was constructed at the southern exit of the bridge. 

### 2.2. Data Collection and Statistical Analysis 

A total of three camera traps were installed to monitor the ecological bridge. Two camera traps were placed at the two-way entrance and exit of the bridge, and a third camera trap was placed overlooking the pond. Camera traps were installed at a height of 30–50 cm from the ground, taking into account the terrain conditions. No bait or attractants were used in the area where the camera traps were installed. The cameras were set to be lag-free, medium-sensitivity, uninterrupted, and with a sequence of five photos per trigger to ensure no animal crossing was missed. The date, time, temperature, and moon condition were also recorded in the camera traps. Between August 2020 and December 2022, 800 days of data were taken in fifteen-day periods. Among the videos and photographs obtained, those containing wild animals were separated, and the type of individual, date, time, temperature, number of individuals, image number, and the state of the moon were processed as data in Microsoft Excel version 16.0. Species at the same site were considered independent when there was an interval of at least 1 h between them [46]. By filtering the image records for each mammal species by a clock, standard descriptors were derived. Thus, multiple images of the same individual standing in front of the camera trap were prevented from being scored as multiple events [47]. The collected data were subjected to statistical analysis using bnlearn [48] and visualized by using ggplot2 [49] packages within the R environment (v4.3.2; [50]). 

### 2.3. Bayesian Network Model

Bayesian networks are a type of probabilistic graphical model used to represent a collection of random variables and their interrelated dependencies using directed acyclic graphs (DAGs). In this graphical representation, each node corresponds to a random variable, and the connections between nodes signify conditional relationships among them [51]. These conditional relationships are described through conditional probability tables (CPTs), which detail the likelihood of each possible value of a node based on the values of its parent nodes. Bayesian networks find application in diverse tasks such as decision making, risk assessment, and prediction [52]. They prove especially valuable in scenarios marked by uncertainty or incomplete data, as they can handle missing or noisy information and make predictions even in the presence of unknown input variables. One of the notable advantages of utilizing Bayesian networks lies in their ability to offer a clear and interpretable means of depicting intricate associations between variables. They can simulate complicated systems and provide insights on how these systems should behave under different scenarios [53].

Let us consider a directed acyclic graph (DAG) denoted as **G** = (**N**, **A**), where **N** represents a finite set of nodes, and **A** represents a finite set of directed arrows connecting these nodes. This DAG serves as the structural representation of a Bayesian network (BN). Each node, represented as “*n*” in **N** within the graph **G**, corresponds to a random variable *Xn*. We denote the set of variables associated with graph **G** as **X** = (*Xn*) ∈ **N**.

For each node with parents, denoted as (*n*), a local probability distribution (*xn*|(*n*)) is assigned. The Bayesian network for the random variables *X* is a combination of the graph **G** and the set of local distributions P (**G**, **P**), where **P** represents the collection of these local distributions for all variables within the network [54]. 

The absence of arrows in graph **G** signifies conditional independence among the random variables *X*, which enables the factorization of the joint probability distribution:

(1) $$p\left(x\right)=\underset{n\in N}{\prod }p(x_{v}\setminus x_{pa\left(v\right)})\;$$

Equation (1) involves a combination of discrete and continuous variables [55]. There are two main categories of algorithms for learning the structure of Bayesian networks. The first type is the search-and-score algorithm, which assigns a score to each possible BN structure and selects the one with the highest score. The second type is known as constraint-based structural learning, which involves performing conditional independence analyses on the data to construct an undirected graph. This undirected graph is then transformed into a BN with the aid of an additional independence test. We implemented the Tabu search algorithm in the analysis. The Tabu search algorithm represents a proficient global optimization approach integrating adaptive memory, facilitating exploration beyond local search to ascertain the global optimum. By employing a “Tabu list” mechanism, this method circumvents repetitive solution iterations while leveraging aspiration criteria to activate favorable solutions. Notably advantageous in resolving global optimization challenges, the Tabu search algorithm has found widespread application across diverse domains in recent years [56]. Thus, we employed Bayesian networks with the Tabu search algorithm to model the wild boar crossings and associated factors. Our aim is to understand the relationship among these factors and their dependencies. In addition, the crossing trend of wild boars was analyzed using Bayesian networks, leading to the identification of four distinct scenarios based on the objectives of the study. These scenarios were constructed using variables such as pond condition, season, lunar phase, date, and time. The scenarios were employed to investigate potential differences in the utilization of these variables between individual and herd crossings. The likelihood of each node based on its parent node is depicted through CPTs. The variables, their descriptions, and the time ranges are given in Table 1.

## 3. Results

As a result of the study, a total of six species of medium and large mammals were observed: golden jackal, red fox, wild boar, European badger, European hare (*Lepus europaeus*), and beech marten (*Martes foina*). From August 2020 to December 2022, these six wild mammal species exhibited a combined total of 686 instances of movement. Among the species captured by camera traps in the study area, the wild boar was the most frequently recorded with 586 images, while the beech marten had the lowest number of records, with just 1 image (0.1%). Following the wild boar, 69 photos are of red foxes, 7 of European hares, 3 of golden jackals, and 2 of European badgers. Wild boar crossings represented 87.7% of all documented data, whereas fox crossings accounted for 10.3%. According to the study findings, 183 of the 586 wild boar crossings occurred in 2020, 254 in 2021, and 149 in 2022. There is a fluctuation in crossing numbers over time. 

The crossings of wild boars, whether herd or individually, are counted as a single crossing. Assessing wild boar crossings based on herd size revealed that more than half of the crossings were those made by herds, with 297 (50.7%) crossings. The remaining 49.3% comprised 289 passages by individual wild boars. The maximal herd size detected of the wild boar crossing the overpass reached 18. Herd sizes ranging from 2 to 5 individuals recorded 199 crossings, while groups of 6 to 10 individuals accounted for 102 crossings. A total of 45 crossings were observed in herds comprising 16 to 18 individuals. Additionally, herds comprising 16 to 18 individuals demonstrated a minimum of 13 crossings.

In 2020, wild boars crossed the bridge 183 (31.2%) times, which increased to 254 (43.3%) in 2021 and decreased to 149 (25.4%). Analysis of wild boar crossings by season revealed that the most crossings occurred during autumn with 318 cases. These autumn crossings accounted for 54.3% of all seasons, followed by 119 (20.3%) passes during summer and 79 (13.4%) passes during winter. The fewest crossings were observed during the spring season, with 70 (11.9%) crossings. 

**Figure 2:**
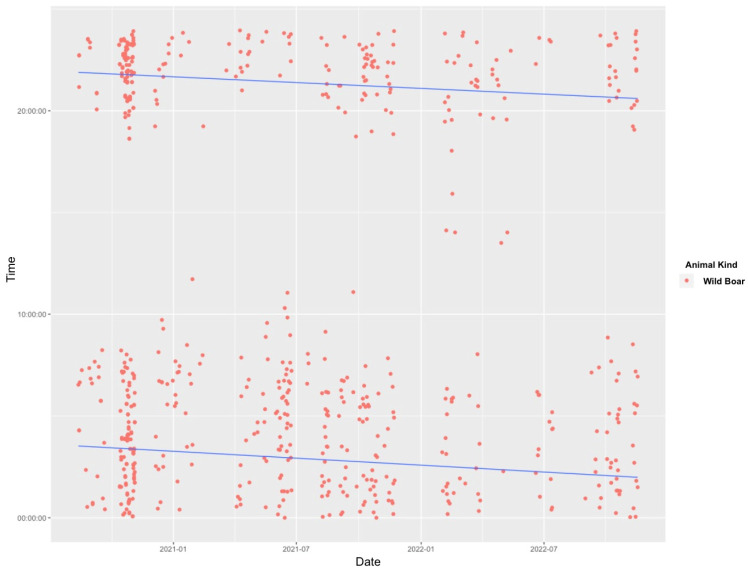
Wild boar crossing trend across various time intervals.

When examining the temporal patterns of wild boar crossings across the overpass, it is noteworthy that these animals exhibit a higher level of activity during crepuscular hours (Figure 2). The most substantial crossing activity, totaling 223, was recorded between 22:00 and 02:00. This time frame accounted for 38.1% of all observed crossings. Subsequently, during the 02:00–06:00 time frame, there were 161 recorded crossings, followed by the 06:00–10:00 period, which accounted for 100 crossings. In contrast, the diurnal period from 14:00 to 18:00 exhibited the least activity, with a mere four recorded crossings. Approximately 80.7% of wild boar crossings occurred between sunset and sunrise. In addition, there were no detections in January.

The crossing trend of wild boars showed that the majority of these crossings concentrated within two distinct lunar phases, which accounted for a substantial 89% of the total crossings. The highest number of crossings occurred during the swollen moon, with 280 crossings (47.8%), followed by 247 crossings (42.2%) during the crescent moon. Notably, a minimal six crossings (1%) were observed during the new moon (Table 2).

**Figure 3:**
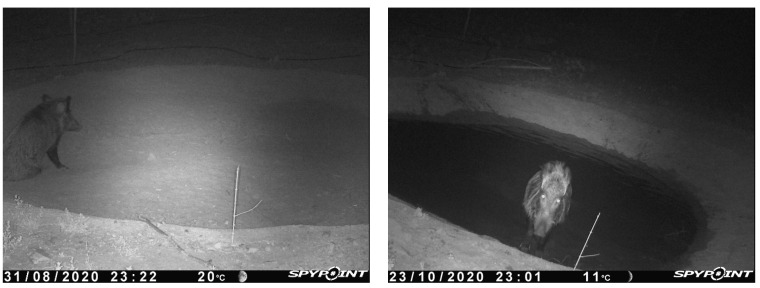
Photographs of wild boars in the pond captured with a camera trap.

Wild boar crossings were observed to take place when the pond in the ecological bridge was at full capacity, amounting to a total of 498 crossings. We considered the pond as full capacity if the water level in the pond was enough for the wild boars to use and as empty capacity if there was no water left in the pond. Crossings when the pond was at full capacity constituted 85% of all crossings, while the remaining 15% (88 crossings) occurred when the pond was empty (Figure 3).

The *t*-test was employed to examine potential differences in variables between herd and individual crossings. As a result, there is a statistically significant difference between herd and individual crossings in terms of seasonal crossings (t = 4.420, df = 584, *p* < 0.05). Similarly, there is a statistically significant difference between herd and individual crossings in terms of crossing periods (t = −3.134, df = 584, *p* < 0.05). We used Bayesian networks to explain how these seasonal movements differ in terms of herd and individual transitions. The greatest number of individual and herd crossings was observed during the autumn season. However, disparities in seasonal transitions were also noted, including variations in time intervals for the least number of crossings. Specifically, the spring season had the fewest occurrences of herd crossings, while the winter season experienced the least frequency of herd crossings. 

**Figure 4:**
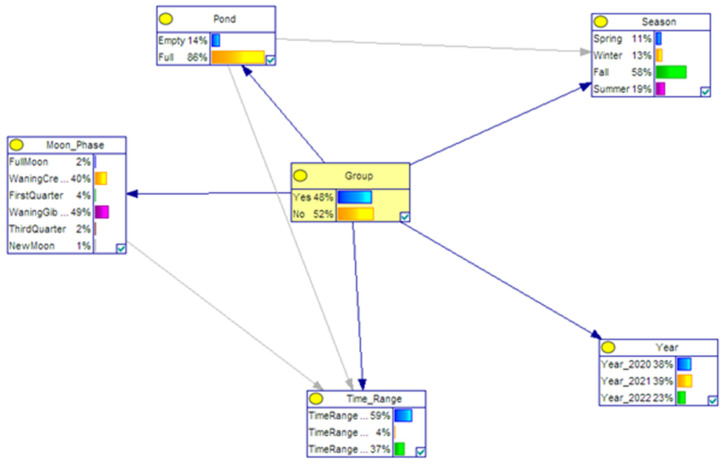
Main conditional probability network.

The crossing patterns of wild boars were analyzed using Bayesian networks, leading to the identification of four distinct scenarios based on the study’s objectives, statistical findings, image examination, and the testing of various models. These scenarios were constructed using variables such as pond condition, season, lunar phase, date, and time. The scenarios were employed to investigate potential differences in the utilization of these variables between individual and herd crossings. The scenarios are given in Table 3. In Table 3, if a categorical criterion is not chosen for the variable, it is represented by “x”. The likelihood of each node based on its parent node is depicted through conditional probability tables (CPTs). Figure 4 and Table 4 serve as an illustration of a CPT.

During the construction of the Bayesian network, three distinct models were developed using different algorithms: TAN, Tabu Search, and Bayesian Search. Subsequently, their predictive performance was evaluated based on logscore values. The algorithm demonstrating a higher logscore indicates a better-performing model. Hence, the model with the highest logscore was chosen. The Tabu Search algorithm yielded the highest logscore of −475.80, suggesting that the model estimated through Tabu Search best fits the data. Following this, the TAN algorithm produced the second-best performance with a logscore of −478.01, and the Bayesian Search algorithm resulted in the third-best performance with a logscore of −479.09. Consequently, the Bayesian network constructed via the Tabu Search algorithm was selected. To understand how changes in other node levels, such as the pond, phase of the moon, year, season, etc., affect crossings, entropy scores for each node are calculated. The crossings are primarily influenced by the year (0.3333). Subsequently, the second most impactful variable is the season (0.125), followed by the pond (0.115), the phase of the moon (0.111), and finally, the time range (0.028).

**Figure 5:**
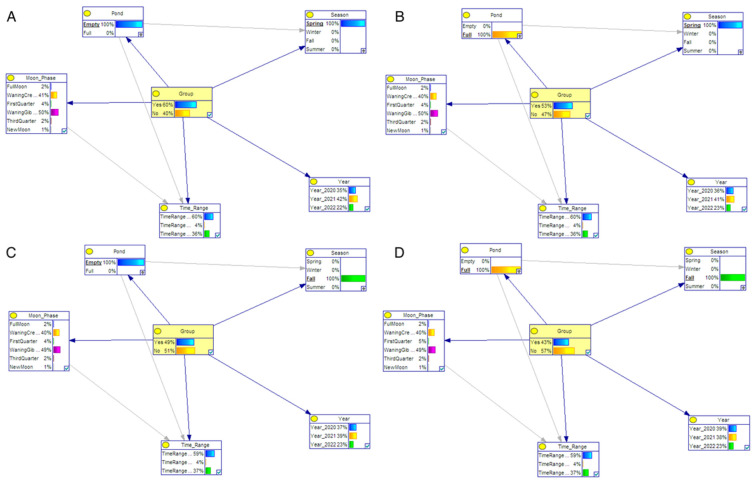
(**A**) First scenario entails an empty pond during the spring season, (**B**) second scenario presents a full pond in the spring season, (**C**) third scenario involves an empty pond during the autumn season, and (**D**) fourth scenario showcases a full pond during the autumn season.

In order to enhance comprehension regarding the influence of pond conditions, seasonal variations, and moon phases on group crossing, a comprehensive analysis comprising four distinct scenarios was conducted. These scenarios were devised to meticulously observe the fluctuations in probabilities associated with group crossing amidst specific conditions influenced by the status of the pond and prevailing seasonal variations. Table 3 shows the independent variables within each scenario. In the first scenario, during the spring season and when the pond was empty, a significant distinction between individual and herd crossings was observed. Herd crossings were estimated to be 1.5 times more likely (60–40%) than individual crossings in this specific scenario (see Figure 5A). In the second scenario, when the pond was full, this difference in probabilities decreased; however, herd crossings still exhibited a higher likelihood compared to individual crossings (see Figure 5B). In the third scenario (see Figure 5C), the probability of crossings occurring during the autumn season when the pond was empty was higher for herds than for individual crossings (48–52). In the fourth scenario, this probability increased even further when the pond was full during the same season (see Figure 5D).

The investigation into how the pond influences the crossing behavior of wild boars, whether individually or herd, revealed insights through Bayesian networks. While the pond did not affect herd crossings, it was found to enhance individual crossings. For instance, in scenario A, during spring when the pond was empty, 40% of crossings were individual; however, this figure rose to 47% when the pond was full. Similarly, in autumn, with an empty pond, 51% of crossings were individual, but this percentage increased to 57% when the pond was full. For this reason, given the current results, we advise against incorporating a pond in the planned bridge design. Furthermore, upon analyzing the seasonal impacts, it was evident that during spring crossings, the majority (60% and 53%) involved herds, whereas in autumn, the trend reversed, and individual crossings (51% and 57%) surpassed herd crossings.

## 4. Discussion

Understanding the specific requirements and behaviors of wildlife species holds significant importance in mitigating wildlife–vehicle collisions (WVCs) and preserving ecological connectivity. Here, the biggest challenge in integrated approaches to wildlife crossing planning is the selection of a single species as the target species [57]. Typically, the selection of vulnerable species as target species is a widely employed method [5]. When aiming to mitigate collisions involving wild boars, it is crucial for these expensive structures to serve the broader purposes of preserving expansive ecosystems and ensuring habitat protection.

Marten, badger, jackal, and rabbit species were rarely seen on the studied ecological bridge during the study period. The fox and wild boar are the most observed species using the bridge. The intense use of the two species may be due to their adaptation to the bridge as well as their higher population densities. Another factor may be that the number of wild boars has increased as a result of the lack of predators in the area where the study area is located. Other species used the ecological passage when the bridge first opened. This period coincided with the time of curfews due to the COVID-19 pandemic. During these periods, night traffic experienced its lowest frequency. Likewise, although the data for 2020 covers the six-month period after August, it constitutes 55% of fox crossings (n = 38) and 37.4% of wild boar crossings. In other words, the wild boar was the most observed species using the bridge, while the fox occurred only sporadically.

While females and cubs of wild boars live in herds, adult males, defined as fierce, live alone. The species has a wide distribution throughout the world and has demonstrated remarkable adaptability to diverse habitat conditions [58], highlighting its strong adaptive capabilities. Wild boars are primarily opportunistic feeders with a highly flexible diet, consuming various plants and animals that can vary based on geographical location and season [59]. This species, particularly the male, is fertile and reproduces vigorously [60]. The ecological bridge serves as a passage for both individual wild boars and herds, but the utilization is not evenly distributed. Notably, in 2020, the majority of crossings were by individual wild boars (n = 115, 19.7%), while in 2021, herd crossings dominated (n = 166, 28.3%). This suggests that in 2020, wild animals may have used the bridge more during restricted human activities due to pandemic-related bans [61,62], while undisturbed herds preferred not to move. Additionally, during the autumn season with a full pond, there was a high probability of ferret activity during sunrise hours, likely influenced by decreased water resources due to the dry period in the Aegean region, prompting a preference for pond usage.

In general, autumn emerges as the primary season during which wild animals, including wild boars, make the most frequent use of the ecological bridge. Several factors contribute to this trend. In wild boars, the coping strategy for seasonal changes seems closely tied to their mobility capabilities [63]. Their increased mobility in autumn may be attributed to a reduction in available agricultural areas for feeding and a minimal decrease in water resources in the region. One motivating factor for this increased mobility is the imperative of avoiding predators [64]. In other regions, shifts in habitat usage occur during both the vegetation period (March–August) and the hunting period (September–December) of wild boars in agricultural areas [64]. As crops mature, wild boars actively engage with agricultural spaces, displaying a preference for feeding near the forest edge [65]. However, in contrast, Thurfjell et al. [65] note that during the autumn season, they avoid the forest edges. The reasons cited for this behavior are both hunting pressure and acclimatization to feeding patterns. Lastly, Lagos et al. [66] observed a distinct peak in WVCs involving wild boars across Europe in autumn, aligning with both the hunting season and the rutting period. This is consistent with our results. 

In natural settings, wild boars commonly exhibit alternating periods of activity and rest throughout both day and night [67]. In human-dominated landscapes, such as the study area, wild boars tend to predominantly move during the night to minimize interactions with humans, irrespective of seasonal changes in photoperiod [68]. Their activity rhythm is characterized as biphasic or polyphasic, displaying high intraspecific variability, with a primary focus on foraging during their active periods [69]. The initiation of their activity is closely linked to sunset [70]. This behavior aligns with existing literature when considering the use of ecological bridges by wild boars, where both wild and herd crossings exhibit similar patterns [71,72].

Our findings show that 38.1% of total crossings occur between 22:00 and 02:00 at night, contrasting sharply with the mere 1.6% of crossings taking place between 10:00 and 18:00 during the day. Seasonal variations are observed, influenced by the duration of darkness. For instance, a decline in crossings is noted between 18:00 and 22:00 and between 14:00 and 18:00 in summer, while an increase is observed during these hours in winter. Research on WVCs suggests that collisions with wild boars are generally linked to sunrise and sunset [28]. Even though the study reveals that the majority of crossings take place at dusk, the chance of encountering wild animals diminishes as the traffic volume decreases to a minimum during these hours. 

One of the most important findings of this study is related to the lunar cycles in wild boar crossings. Wild boar crossings were most frequent during the Waxing Gibbous and Waxing Crescent, comprising 49.5% and 40.5% of the crossings, respectively. The least crossing occurred during the new moon phase with 1.1%. This trend is likely attributed to the increased brightness during these phases, which can reduce the nocturnal activity of ungulates, making them more vulnerable to detection by predators [73,74]. Despite wild boars having poor eyesight, their observed significant reduction in activity during brighter nights [75,76] aligns well with the findings of the study. The association between the lowest brightness during the new moon phase, when ecological bridge crossings by wild boars are least frequent, can be attributed to the impaired vision of wild boars during this phase. Examining the alignment of wild boar movements with the moon provides valuable insights into the potential for wildlife–vehicle collisions. Importantly, the increased frequency of ecological bridge crossings during specific monthly periods does not necessarily imply a corresponding rise in WVCs during those times. Limited information exists about the frequency of WVCs concerning the lunar cycle, in Türkiye and even around the world [77]. Colino-Rabanal et al. [78] investigated the effect of moon phases on ungulate–vehicle collisions and identified a strong positive relationship between wild boar and vehicle collisions and moon brightness, particularly during the full moon. When crossings are higher, the probability of collision may increase. For instance, if the lowest number of crossings occur at full moon and the number of collisions is high, there can be another variable such as driver behavior that is triggered by the full moon. Overall, there was no effect of the phases of the moon on herd or individual crossings. 

Ecological bridges are strategically constructed along highways at regular intervals. Larger species, such as the wild boar, broaden their movements in fragmented environments and often navigate through anthropogenic matrices to connect various fragments. This is because small habitats cannot sustain sufficient resources to support populations of these animals [79]. The home range stands out as perhaps the most reliable indicator of normal animal movement [80]. Utilizing home range data, cluster analysis can identify species with similar movement dynamics and group them based on home range scales [81]. Given the high cost associated with wildlife overpasses, their construction should be limited to very special cases. Considering the constraints of limited resources, the primary objective of transportation and conservation planning or management is to implement conservation actions that offer the greatest benefit and are economically optimal [82]. According to a study, it was emphasized that acquiring agricultural land for nature restoration is more cost-effective than constructing wildlife crossings [83].

## 5. Conclusions

The primary consideration concerning the ecological bridge constructed on the Izmir-Çeşme motorway is its necessity. The occurrence of wild boar fatalities on the road was frequent prior to the construction of the bridge. On the contrary, throughout the observation period, there were no instances of wild boar fatalities subsequent to the completion of the bridge. However, from our field experience, we observed that even though the Izmir-Çeşme motorway is enclosed by wire fences, wild boars still find a way to enter the highway by tearing through these wires in specific areas. This may result in WVCs. Although this paper does not aim to investigate cost-effective methods for wildlife crossing, there is a need to assess the feasibility of preventing the passage of wild boars at a lower cost. 

It is crucial to enhance the utilization of the existing ecological bridge by wild animals. Given the typical lifespan of wildlife crossing structures, which is approximately 70–80 years, it is imperative that the location and design of such crossings evolve to accommodate the dynamic changes in habitat, climate conditions, and wildlife populations over time. Initially, common regional species like rock marten, rabbit, jackal, and badger successfully utilized the overpass upon its opening. However, there is a lack of evidence to confirm the continued presence of these wild animals in later times. 

The study highlighted a significant observation regarding the impact of water resources on wild boar movements. Wild boars may cross highways for two major reasons: food and water sources. Agricultural areas, particularly in Uzunkuyu and Nohutalan Districts to the north of the bridge and Zeytineli District to the south, serve as a food source for wild boars. However, the region faces a potential challenge of water scarcity, a key finding emphasized in the study. One of the most important findings of the study is related to the use of a pond by wild boars. The use of a pond by wild boars varies from June, when the rain decreases or ceases, until December. Here, the three outputs we obtained from the camera trap images are very important. The first of these is the differentiation between herd use and individual use, the second is the increase in the time spent by individuals in the pond, and the last one is the competition among wild boars to access the pond. Our model results showed that individual use was higher than herd use when the pond was full in the fall. In fact, the question that needs to be answered here is, can increasing artificial water resources reduce the crossing of wild boars on highways? Another question can be how far these resources should be placed/created from the highways to keep boars away from crossing and not aggregate them near the danger zone.

While this study does not specifically address pond design or issues related to ponds, the question of whether ponds are crucial in significant ecological overpasses remains a separate topic open to debate. Improper pond design can cause diseases among animals [84]. A significant concern associated with ponds is the potential for the transmission of diseases among wild boars. Implementing measures to prevent the spread of diseases among wild boars and to other wildlife species is crucial. Wild boars act as reservoirs for various viral and bacterial diseases, as well as parasites [85]. Many of these diseases and parasites pose risks to humans, livestock, and other wildlife. Ponds often serve as cooling mechanisms, with male wild boars spending extended periods, particularly during the summer months. The extended presence of male individuals in the pond emphasizes the need for more frequent water changes or the implementation of automatic water systems.

The implementation of wildlife crossing structures enhances traffic safety by enabling animals to move safely on roads, thereby contributing to biodiversity conservation and decreasing the likelihood of wildlife–vehicle collisions. Our results suggest that wild boars have actively used the bridge since its opening. This shows that wild boars can adapt to these crossings, and therefore, we recommend the implementation of wildlife crossings to facilitate ecological connectivity for wild boar populations. Thus, wildlife crossings can maintain the flow of genes and contribute to wildlife conservation. In Türkiye, further study is needed to gain a deep understanding for determining the integration between ecological connectivity and WVCs. The findings of this study can be used as an adaptive guide for the design of future wildlife crossing structures, with a focus on target wildlife species.

## Figures and Tables

**Table 1:** Description of the variables.

Variable	Description	Categories
Group	Herd crossings were categorized as “yes” and individual crossings as “no”.	Yes
		No
Pond	If there is water in the pond, it is categorized as “full”, and if there is no water, it is categorized as “empty”.	Empty
		Full
Season	The 4 different seasons (fall, winter, spring, summer) in Türkiye are categorized.	Spring
		Winter
		Fall
		Summer
Year	The three different years in the dataset categorized as 2020, 2021, and 2022.	2020
		2021
		2022
Time Range	Three different time ranges based on the wild boar crossing are categorized.	00.00–08.00 (Time_Range1)
		08.00–16.00 (Time_Range2)
		16.00–00.00 (Time_Range3)
Moon Phase	Six different phases of the moon are categorized according to dataset.	Full Moon
		Waning Crescent
		First Quarter
		Waning Gibbous
		Third Quarter
		New Moon

**Table 2:** The frequency of wild boar crossings according to the moon phases.

Moon’s Phase	Frequency of Crossing	Percentage	Cumulative P.
New Moon	6	1.0	1.0
Waning Crescent	247	42.2	43.2
First Quarter	25	4.3	47.4
Waning Gibbous	280	47.8	95.2
Full Moon	15	2.6	97.8
Third Quarter	13	2.2	100.0
Total	586	100.0	

**Table 3:** Description of the scenarios.

Scenario	Pond	Season	Phase of Moon	Time Range	Year
1	Empty	Spring	x	x	x
2	Full	Spring	x	x	x
3	Empty	Fall	x	x	x
4	Full	Fall	x	x	x

**Table 4:** Conditional probability table.

Variable	Categories	Probabilities
Group	Yes	48%
	No	52%
Pond	Empty	14%
	Full	86%
Season	Spring	11%
	Winter	13%
	Fall	58%
	Summer	19%
Year	2020	38%
	2021	39%
	2022	23%
Time Range	00.00–08.00	59%
	08.00–16.00	4%
	16.00–00.00	37%
Moon Phase	Full Moon	2%
	Waning Crescent	40%
	First Quarter	4%
	Waning Gibbous	49%
	Third Quarter	2%
	New Moon	1%

## Data Availability

The data presented in this study are available on request from the corresponding author. The data are not publicly available due to privacy.
